# Stockading a Loose Cartilage with Hare-Lip Pins to Fix It for Operation

**Published:** 1883-01

**Authors:** Edmund Andrews

**Affiliations:** Professor of Surgery in Chicago Medical College; No. 6 Sixteenth St., Chicago


ARTICLE V.

STOCKADING A LOOSE CARTILAGE WITH HARE-LIP PINS TO FIX IT FOR OPERATION. By EDMUND ANDREWS, M.D., Professor of Surgery in Chicago Medical College.

A patient came to me from Dakota with a loose cartilage in the knee-joint. There was the usual complaint of its catching suddenly in the articulation so as to produce violent pain, followed by synovitis, and reason to fear that, by continued repetition of these accidents, destructive disease of the knee would be set up.

The object was of moderate size, and had this peculiar behavior, that it could not be cornered in the usual pocket in the antero-lateral fold of the synovial membrane where it laps over the edge of the tibia. Instead of this, whenever it appeared within reach, it was in the broad fold of the sac, where it laps upward on the side of the condyle of the femur. I found the space here was so wide that I could not retain the slippery cartilage in one spot long enough to extract it, for with the slightest change in the position of the fingers, I lost it into the interior of the articulation.

After being baffled in this way three or four times, I attempted to transfix the body with a cataract needle, but found it calcified, and so hard that I only bent the point of the needle, while a stronger needle, next resorted to, would not penetrate the object enough to get a secure hold.

Defeated in all these efforts, I next took a number of hare-lip pins, and inserted them down to the condyle in a circle around the floating body, and thus confined it in a stockade. Then, with a tenotome and a pair of forceps, I succeeded without difficulty in the extraction.

The operation was done under carbolized spray, and with all antiseptic precautions. A very slight synovitis ensued, but it was insignificant, and subsided without any evil results.

*No. 6 Sixteenth St., Chicago.*

